# Meat, vegetable, and fruit consumption among urban and rural elders aged 60+ years in regional China: a population-level nutritional study

**DOI:** 10.1017/jns.2023.104

**Published:** 2023-11-30

**Authors:** Guilin Zhang, Jian Kang, Shibao Jing, Yinhao Chen, Tianrui Deng, Huiqing Xu, Haidi Wu, Fei Xu

**Affiliations:** 1 Geriatric Hospital of Nanjing Medical University, Nanjing, China; 2 The First Affiliated Hospital with Nanjing Medical University, Nanjing, China; 3 Nanjing Liuhe District Center for Disease Control and Prevention, Nanjing, China; 4 Wenzhou Center for Disease Control and Prevention, Wenzhou, China; 5 Nanjing Medical University School of Public Health, Nanjing, China; 6 Nanjing Municipal Center for Disease Control and Prevention Affiliated to Nanjing Medical University, Nanjing, China

**Keywords:** Elders, Fruit, Intake, Red meat, Vegetable, White meat

## Abstract

The aim was to assess epidemiological characteristics of the most recent consumption patterns of meat, vegetable, and fruit among representative urban and rural residents aged 60+ years in regional China. In this cross-sectional survey conducted in mid-2018, participants aged 60+ years were randomly chosen from urban and rural communities in Nanjing municipality of China. Meat, vegetable, and fruit intake were assessed with a validated food frequency questionnaire. Multivariate logistic regression models were applied to compute odds ratio (OR) and 95 % confidence interval (CI) to investigate the association of socio-demographic characteristics with a likelihood of meeting intake recommendation. Among the 20 867 participants, 49⋅5 % were men and 45⋅0 % urban elders, and 6⋅5 % aged 80+ years. The mean values of consumption frequency of red meat, white meat, vegetable, and fruit were 2⋅99 ± 2⋅28, 1⋅37 ± 1⋅13, 5⋅24 ± 6⋅43, and 2⋅64 ± 2⋅91 times/week, respectively, among overall participants. Moreover, there were 14⋅9, 23⋅7, and 12⋅1 % of participants meeting intake recommendations of meat, vegetable, and fruit, separately, in this study. After adjustment for potential confounders, age, gender, residence area, and educational attainment each was associated with the likelihood of meeting intake recommendation of meat, vegetable, or fruit. The consumption frequency and proportion of participants meeting intake recommendations of meat, vegetable, or fruit were not high among elders in regional China. Socio-demographic characteristics were associated with intake recommendations of meat, vegetables, and fruit. It has public health implications that participants’ socio-demographic attributes shall be considered for precision intervention on meat, vegetable, and fruit consumption in healthy eating campaigns among elders in China.

## Introduction

Meat, vegetable, and fruit, the major types of daily foods, are essential to human health. It has been well documented that inadequate consumption of meat, vegetable, and fruit is associated with the risk of experiencing non-communicable diseases (NCDs).^(1–8)^ Unbalanced consumption of meat was associated with type 2 diabetes (T2D), cardiovascular diseases (CVDs), and some cancers,^(1–4)^ while inadequate intake of vegetable and fruit has been examined in relation to T2D and CVDs as well as mental health issues.^(5–8)^ Moreover, unhealthy consumption of meat, vegetable, and fruit has produced a heavy health-related burden globally based on premature mortality.^(9,10)^ Therefore, healthy eating of meat, vegetable, and fruit is an important public health concern worldwide. And, consequently, periodical investigation on and population-level evaluation of meat, vegetable, and fruit consumption are of particular significance for precision interventions on community-based NCDs through healthy eating promotion from the perspectives of both nutritional epidemiology and public health.

Systems of such a periodical surveillance on meat, vegetable, and fruit consumption have been established among the overall population as a part of the Behavioral Risk Factor Surveillance Systems (BRFSS) in some countries/regions by specific official institutes (e.g., US CDC, China CDC).^(11,12)^ In addition to these information collected dynamically from the general population by official institutes, specific data on meat, vegetable, and fruit consumption were also gathered among different sub-populations by academic researchers from different countries/communities.^(13–18)^ Particularly, some studies reported meat, vegetable, and fruit consumption with a focus on elders, a particularly vulnerable sub-population, using data derived from the overall adult population.^(19–23)^

With a rapid transition to an ageing society, China has been witnessing a remarkable rise in the prevalence of NCDs among elders aged 60+ years over the past decades.^(24–26)^ Moreover, China is a so big country that its geographic areas and economic growth are with diversity. Residents’ lifestyle/behaviour patterns differ in regions/communities at different economic development stages in China. Therefore, for the purpose of precision prevention of NCDs through eating behaviour intervention among elders, it is of continuous interest to investigate and assess the consumption of meat, vegetable, and fruit, the main modifiable eating behaviours, not only nation-wide but also region-specifically in China. For this purpose, we conducted a study with the aim of investigating the most recent intake level and epidemiological characteristics of meat, vegetable, and fruit among representative urban and rural residents aged 60+ years in regional China.

## Methods

### Study design and participants

Data analysed in the present study were from a broad cross-sectional survey (Healthy Aging, Healthy Elders 2018, HAHE-2018 study), which was conducted to collect information on common NCDs (including T2D, hypertension, low/excess body weight, etc.), participant's lifestyle and behaviours (including eating behaviours, physical activity, etc.) among local urban and rural residents aged 60+ years in Nanjing municipality of China during mid-2018. Subjects were eligible for taking part in the HAHE-2018 study, if they (1) were locally registered inhabitants aged 60+ years, (2) did not have physical/psychiatric problems, and (3) did not have literal/cognitive problems. The sample size of HAHE-2018 study was estimated based on consideration of (1) the multi-stage sampling approach applied in the survey, (2) the lowest prevalence of common NCDs (1⋅4 %, the prevalence of underweight among men aged 60+ years) among elders in China,^(27)^ (3) the response rate (85 %) expected, and (4) the statistical power (90 %) assumed. Thus, about 21 000 participants would warrant a statistical power for identifying the sufficient number of elders with low body weight in HAHE-2018 study. Consequently, such a large number of subjects would be more than that required for investigating the consumption of meat, vegetable, and fruit, as either intake rate of these three types of food was really much higher than the figure of low body weight among elders in China.^(21,22)^

Nanjing, the study municipality, is a typical megacity in eastern China, which had approximately 8⋅43 million registered inhabitants with about 18⋅0 % of elders aged 60+ years in 2018.^(28)^ To maximize the representativeness of the general population of elderly people, a multi-stage sampling strategy was used to randomly recruit subjects from each of the twelve administrative districts of Nanjing. When the overall sample size was determined, the number of eligible participants was computed for each district according to the specific district-to-municipality proportion of elders. Next, the number of potentially participating households was calculated for each district based on an assumption that two elders from one household would be recruited in the survey. Then, a two-stage sampling approach was applied to select participants. Firstly, fifteen communities/neighborhoods were randomly selected from each district. Secondly, participating households were randomly recruited from each selected community/neighborhood based on the number of households estimated for the district. Finally, all eligible elders within determined households were invited to take part in the investigation. Thus, 20 883 eligible participants were successfully recruited in the HAHE-2018 study.

All participants were asked to provide their written informed consent prior to the implementation of the HAHE-2018 study. Although data used to describe epidemiological characteristics of meat, vegetable, and fruit consumption were from HAHE-2018 study without the personal identification of participants, the present study was also reviewed and approved by The Ethics Committee of Geriatric Hospital of Nanjing Medical University. The methods performed in the study were in line with recommendations by the Declaration of Helsinki.

### Data collection

Participant's personal socio-demographic characteristics (age, residence area, educational attainment, marital status), history of common NCDs (hypertension, diabetes, abnormal lipid profile), dietary consumption (intake of red and white meat, vegetable, and fruit), physical activity, cigarette smoking and drinking, family history of hypertension and diabetes were self-reported with a widely-used standardized questionnaire via a face-to-face interview administered by investigators.^(29)^ Participants’ weight and height were objectively measured with light clothing and no shoes. Body weight was assessed to the nearest 0⋅1 kg, while height was recorded to the nearest 0⋅01 m. Each of them was measured twice for each participant and the mean values were used to calculate body mass index (BMI) for each subject in the study.

### Study variables

#### Outcome variable

Meat (red and white meat, separately), vegetable, and fruit consumption was assessed using a validated Chinese version of the food frequency questionnaire (FFQ).^(30)^ The consumption frequency (times/week) of these foods was, one by one, recorded for each participant, as consumption frequency more than intake amount was easier to be recalled and self-reported.^(11)^ The question in the FFQ used to collect information on food consumption was ‘In last year, how often did you consume the following foods under a typical situation? Please respond to all items of food in frequencies one by one.’.^(30)^ Then, the consumption frequencies were converted into times/week for analysis. Further, the mean values (standard deviation, sd) of red meat, white meat, vegetable, and fruit consumption frequencies were calculated for each participant in the study. Chinese Nutrition Society in 2016 released a recommendation on consumption frequencies of meat, vegetable, and fruit specifically for Chinese elders.^(31)^ In this dietary recommendations for Chinese elders, red meat and white meat were not differentiated from each other.^(31)^ Thus, consequently, the term ‘meat’ referred to the combination of red meat and white meat regarding recommendations on meat consumption for participants in this study. Based on this recommendation, participants were classified into two categories: recommendation reached (‘Yes’) or recommendation not reached (‘No’) regarding meat (red and white meat combined), vegetable and fruit consumption, separately, in the analysis.

### Explanatory variables

Some classical variables were involved in classifying participants for the analysis. Participants’ socio-demographic attributes referred to age (60–69, 70–79, or 80+ years old), gender (men or women), residence area (urban or rural), educational attainment (≤9, 10–12, or 13+ schooling years), and marital status (single or married/having a partner). It is not easy to define urban and rural areas in China. To ensure nation-level comparability of the definitions of urban and rural areas, China National Bureau of Statistics issued an official rules for categorizing urban and rural areas for statistical use in China.^(32)^ In this study, urban and rural areas were determined using the official categories by the National Bureau of Statistic.

Subjects were also categorized as ‘smokers’ or ‘non-smokers’ and ‘drinkers’ or ‘non-drinkers’, separately, for analysis based on the definitions released by China CDC.^(33)^ Participants’ body weight status was assessed using BMI. Each participant was classified as excess weight (BMI ≥ 24) or non-excess weight (BMI < 24) in the analysis according to BMI cutoffs recommended for Chinese adults by Ministry of Health of China.^(34)^ Additionally, underweight was defined as BMI < 18 in the study.^(34)^

Positive family history (‘Yes’) of hypertension and diabetes referred to that either father or mother has been diagnosed as a hypertensive or diabetic patient, respectively. Otherwise, a negative family history (‘No’) of hypertension or diabetes was recorded. A participant was classified as a hypertensive or diabetic patient (‘Yes’) if he/she self-reported ever being diagnosed as a patient by a registered physician. As for the status of the lipid profile, a subject was categorized as having an abnormal lipid profile in the analysis, if he/she has been identified as abnormal with either cholesterol, triglyceride, or high/low-density lipoprotein.

### Data analysis

Percentages (%), mean values, and standard deviation (Mean ± sd) were used to describe the food intake frequency distribution within subjects by socio-demographic characteristics, and chi-square and ANOVA tests were applied to examine the difference in meat, vegetable, and fruit consumption frequency and mean values between participants’ sub-groups accordingly. With adjustment for age, gender, residence area, educational attainment, marital status, body weight, smoking, drinking, family history hypertension/diabetes, hypertension, diabetes, abnormal lipid profiles, multivariate logistic regression models were introduced to calculate odds ratios (ORs) and 95 % confidence intervals (CIs) for identifying the associations of participants’ socio-demographic characteristics with meat, vegetable, and fruit consumption. The significance level was *P* < 0⋅05 (two-sided). EpiData 3⋅1 (The EpiData Association 2008, Odense, Denmark) and SPSS version 20⋅0 for Windows (SPSS Inc., Chicago, IL, USA) were used to enter and analyse data, separately.

## Results

In total, 20 883 elders aged 60+ years were recruited for HAHE-2018 study. Of them, 20 867 reported complete information on food consumption and thus were analysed for investigating meat, vegetable, and fruit intake in this study. Among these participants included in the analysis, 49⋅5 % were men and 45⋅0 % lived in urban areas, while 65⋅7 and 6⋅5 % aged 60–69 and 80+ years, respectively. The majority (74⋅5 %) received less than nine schooling years of education, and only 8⋅0 % obtained college-level educational attainment. There were 13⋅1 % of participants who were obese and 2⋅5 % were underweight. Moreover, among participants, differences in genders by age, residence area, or education were statistically significant, separately (Table 1).

**Table 1.** Selected characteristics of participants aged 60+ years in Nanjing, China

	Participants, *n* (%)			*χ*^2^	*P* value^a^
	Overall	Men	Women		
Total	20 867	10 326 (49⋅5)	10 541 (50⋅5)		
Age (years)					
60–69	13 710 (65⋅7)	6630 (48⋅4)	7080 (51⋅6)		
70–79	5800 (27⋅8)	2997 (51⋅7)	2803 (48⋅3)	20⋅29	<0⋅001
80+	1357 (6⋅5)	699 (51⋅5)	658 (48⋅5)		
Residence area					
Urban	9381 (45⋅0)	4539 (48⋅4)	4842 (51⋅6)	8⋅25	0⋅004
Rural	11 486 (55⋅0)	5787 (50⋅4)	5699 (49⋅6)		
Educational attainment (schooling years)					
9−	15 544 (74⋅5)	7053 (45⋅4)	8491 (54⋅6)		
10–12	3647 (17⋅5)	2139 (58⋅7)	1508 (41⋅3)	449⋅15	<0⋅001
13+	1676 (8⋅0)	1134 (67⋅7)	542 (32⋅3)		
Body weight status^b^					
Underweight	523 (2⋅5)	271 (51⋅8)	252 (48⋅2)		
Normal	9262 (44⋅4)	4653 (50⋅2)	4609 (49⋅8)	58⋅18	<0⋅001
Overweight	8355 (40⋅0)	4237 (50⋅7)	4118 (49⋅3)		
Obese	2727 (13⋅1)	1165 (42⋅7)	1562 (57⋅3)		

a Chi-square test.

b Body weight status was categorised based on recommendations for Chinese adults using body mass index.

Table 2 presents the consumption level of red meat, white meat, vegetable, and fruit by selected characteristics of participants aged 60+ years in this study. Overall, 0⋅9 % (*n* 193) and 3⋅3 % (*n* 689) of participants, separately, did not consume red meat and white meat, while only 0⋅1 % (*n* 21) and 3⋅7 % of elders (*n* 776) reported not eating vegetable and fruits, respectively. The mean values of consumption frequency of red meat, white meat, vegetable, and fruit were 2⋅99 ± 2⋅28, 1⋅37 ± 1⋅13, 5⋅24 ± 6⋅43 and 2⋅64 ± 2⋅91 times/week, respectively, among overall participants. The mean intake frequencies of these foods differed, separately, by age, gender, residence area, and educational attainment among participants.

**Table 2.** Consumption level of red meat, white meat, vegetable, and fruit by selected characteristics of participants aged 60+ years in this study

	Number of participants	Intake frequency											
		Red meat			White meat			Vegetable			Fruit		
		Times/week (SD^a^)	*F*	*P* value^b^	Times/week (SD^a^)	*F*	*P* value^b^	Times/week (SD^a^)	*F*	*P* value^b^	Times/week (SD^a^)	*F*	*P* value^b^
Overall	20 867	2⋅99 ± 2⋅28			1⋅37 ± 1⋅13			5⋅24 ± 6⋅43			2⋅64 ± 2⋅91		
Age (years)													
60–69	13 710	3⋅10 ± 2⋅31			1⋅40 ± 1⋅14			5⋅17 ± 6⋅38			2⋅69 ± 2⋅90		
70–79	5800	2⋅81 ± 2⋅23	42⋅05	<0⋅001	1⋅29 ± 1⋅10	25⋅30	<0⋅001	5⋅30 ± 6⋅54	2⋅29	0⋅101	2⋅94 ± 2⋅45	6⋅50	0⋅002
80+	1357	2⋅73 ± 2⋅18			1⋅27 ± 1⋅08			5⋅53 ± 6⋅51			2⋅45 ± 2⋅90		
Gender													
Men	10 326	3⋅08 ± 2⋅29	28⋅45	<0⋅001	1⋅41 ± 1⋅16	27⋅66	<0⋅001	5⋅14 ± 6⋅38	4⋅30	0⋅038	2⋅55 ± 2⋅83	22⋅83	<0⋅001
Women	10 541	2⋅91 ± 2⋅27			1⋅32 ± 1⋅10			5⋅32 ± 6⋅48			2⋅74 ± 2⋅98		
Residence area													
Urban	9381	3⋅59 ± 2⋅56	1255⋅61	<0⋅001	1⋅20 ± 1⋅36	131⋅68	<0⋅001	5⋅73 ± 6⋅02	99⋅69	<0⋅001	3⋅37 ± 3⋅47	1123⋅91	<0⋅001
Rural	11 486	2⋅50 ± 1⋅89			1⋅28 ± 1⋅06			4⋅83 ± 6⋅72			2⋅05 ± 2⋅19		
Educational attainment (schooling years)													
9-	15 544	2⋅80 ± 2⋅19			1⋅31 ± 1⋅10			5⋅46 ± 6⋅65			2⋅50 ± 2⋅76		
10–12	3647	3⋅54 ± 2⋅44	226⋅87	<0⋅001	1⋅54 ± 1⋅22	69⋅40	<0⋅001	4⋅37 ± 5⋅59	43⋅60	<0⋅001	2⋅97 ± 3⋅15	84⋅60	<0⋅001
13+	1676	3⋅61 ± 2⋅49			1⋅49 ± 1⋅16			5⋅04 ± 5⋅95			3⋅29 ± 3⋅62		

a SD, standard deviation.

b ANOVA test.

Table 3 displays the proportion of participants who reached the intake recommendation of meat, vegetable, and fruit for residents aged 60+ years in this study. Overall, there were 14⋅9, 23⋅7,, and 12⋅1 % of participants who met consumption recommendations of meat, vegetable, and fruit, respectively, in this study. The difference in the proportions of participants meeting meat, vegetable, or fruit intake recommendation was examined by each of gender, residence area, and education. However, a significant difference in the proportions of participants between age-groups was observed only for meat consumption in the study.

**Table 3.** Proportion of participants meeting intake recommendation of meat, vegetable, and fruit for residents aged 60+ years in this study

	Number of participants	Proportion of participants who reached intake recommendations for Chinese elders								
		Meat^a^ (red meat + white meat)			Vegetable			Fruit		
		*n* (%)	*χ*^2^	*P* value^b^	*n* (%)	*χ*^2^	*P* value^b^	*n* (%)	*χ*^2^	*P* value^b^
Overall	20 867	3099 (14⋅9)			4950 (23⋅7)			2527 (12⋅1)		
Age (years)										
60–69	13 710	2209 (16⋅1)	52⋅29	<0⋅001	3202 (23⋅4)	3⋅53	0⋅171	1708 (12⋅5)	4⋅67	0⋅097
70–79	5800	738 (12⋅7)			1406 (24⋅2)			660 (11⋅4)		
80+	1357	152 (11⋅2)			342 (25⋅2)			159 (11⋅7)		
Gender										
Men	10 326	1669 (16⋅2)	27⋅82	<0⋅001	2382 (23⋅1)	4⋅83	0⋅028	1129 (10⋅9)	26⋅58	<0⋅001
Women	10 541	1430 (13⋅6)			2568 (24⋅4)			1398 (13⋅3)		
Residence area										
Urban	9381	1948 (20⋅8)	471⋅40	<0⋅001	2339 (24⋅9)	13⋅83	<0⋅001	2159 (23⋅0)	1904⋅03	<0⋅001
Rural	11 486	1151 (10⋅0)			2611 (22⋅7)			368 (3⋅2)		
Educational attainment (schooling years)										
9-	15 544	2002 (12⋅9)	188⋅24	<0⋅001	3964 (25⋅5)	116⋅27	<0⋅001	1562 (10⋅0)	270⋅89	<0⋅001
10–12	3647	740 (20⋅3)			631 (17⋅3)			603 (16⋅5)		
13+	1676	357 (21⋅3)			355 (21⋅2)			362 (21⋅6)		
Marital status										
Single	4455	568 (12⋅7)	19⋅78	<0⋅001	1877 (42⋅1)	1061⋅07	<0⋅001	562 (12⋅6)	1⋅36	0⋅244
Married/having a partner	16 412	2531 (15⋅4)			3073 (18⋅7)			1965 (12⋅0)		
Smoking										
No	16 048	2263 (14⋅1)	30⋅89	<0⋅001	3988 (24⋅9)	48⋅93	<0⋅001	2032 (12⋅7)	19⋅89	<0⋅001
Yes	4819	836 (17⋅3)			962 (20⋅0)			495 (10⋅3)		
Drinking										
No	16 155	2298 (14⋅2)	22⋅21	<0⋅001	3931 (24⋅3)	14⋅78	<0⋅001	2119 (13⋅1)	68⋅11	<0⋅001
Yes	4712	801 (17⋅0)			1019 (21⋅6)			408 (8⋅7)		

a Meat refers to either red meat or white meat.

b Chi-square test.

Table 4 shows the association of socio-demographic characteristics with the likelihood of meeting intake recommendation of meat, vegetable, and fruit for residents aged 60+ years in the study. After adjustment for potential confounders, age was negatively associated with the likelihood of meeting consumption recommendation for either of meat, vegetable, and fruit among participants in the study. Women were less likely to meet the consumption recommendation of either meat or vegetable compared to men, while no difference in the likelihood of meeting intake recommendation of fruit between women and men (OR = 0⋅93; 95 %CI = 0⋅83, 1⋅03). Moreover, rural elderly people were at significantly lower odds for meeting consumption recommendations of meat, vegetable, and fruit, separately, relative to their urban counterparts. Further, educational attainment were in a positive relation to the likelihood of reaching consumption recommendation of meat but in a negative link to that of vegetable, while only those with college-level educational attainment tended to meet the intake recommendation of fruit (OR = 1⋅15; 95 %CI = 1⋅01, 1⋅33).

**Table 4.** The association of socio-demographic characteristics with a likelihood of meeting intake recommendation of meat, vegetable, and fruit for residents aged 60+ years in this study

	Number of participants	Adjusted OR (95 %CI) for participants to meet consumption recommendations for Chinese elders^a^					
		Meat^b^		Vegetable		Fruit	
		*n* (%)	OR (95 %CI)	*n* (%)	OR (95 %CI)	*n* (%)	OR (95 %CI)
Overall	20 867	3099 (14⋅9)		4950 (23⋅7)		2527 (12⋅1)	
Age (years)							
60–69	13 710	2209 (16⋅1)	1	3202 (23⋅4)	1	1708 (12⋅5)	1
70–79	5800	738 (12⋅7)	0⋅77 (0⋅70, 0⋅84)	1406 (24⋅2)	0⋅91 (0⋅84, 0⋅98)	660 (11⋅4)	0⋅86 (0⋅77, 0⋅95)
80+	1357	152 (11⋅2)	0⋅65 (0⋅54, 0⋅78)	342 (25⋅2)	0⋅70 (0⋅61, 0⋅81)	159 (11⋅7)	0⋅76 (0⋅63, 0⋅92)
Gender							
Men	10 326	1669 (16⋅2)	1	2382 (23⋅1)	1	1129 (10⋅9)	1
Women	10 541	1430 (13⋅6)	0⋅87 (0⋅79, 0⋅96)	2568 (24⋅4)	0⋅78 (0⋅72, 0⋅84)	1398 (13⋅3)	0⋅93 (0⋅83, 1⋅03)
Residence area							
Urban	9381	1948 (20⋅8)	1	2339 (24⋅9)	1	2159 (23⋅0)	1
Rural	11 486	1151 (10⋅0)	0⋅44 (0⋅41, 0⋅48)	2611 (22⋅7)	0⋅58 (0⋅54, 0⋅63)	368 (3⋅2)	0⋅10 (0⋅09, 0⋅12)
Educational attainment (schooling years)							
9-	15 544	2002 (12⋅9)	1	3964 (25⋅5)	1	1562 (10⋅0)	1
10–12	3647	740 (20⋅3)	1⋅24 (1⋅12, 1⋅37)	631 (17⋅3)	0⋅56 (0⋅51, 0⋅62)	603 (16⋅5)	0⋅93 (0⋅83, 1⋅04)
13+	1676	357 (21⋅3)	1⋅25 (1⋅09, 1⋅43)	355 (21⋅2)	0⋅71 (0⋅62, 0⋅81)	362 (21⋅6)	1⋅15 (1⋅01, 1⋅33)
Marital status							
Single	4455	568 (12⋅7)	1	1877 (42⋅1)	1	562 (12⋅6)	1
Married/having a partner	16 412	2531 (15⋅4)	0⋅92 (0⋅83, 1⋅02)	3073 (18⋅7)	0⋅28 (0⋅26, 0⋅31)	1965 (12⋅0)	0⋅51 (0⋅45, 0⋅57)
Body weight status^c^							
Underweight	523	73 (14⋅0)	1	142 (27⋅2)	1	34 (6⋅5)	1
Normal	9262	1347 (14⋅5)	0⋅97 (0⋅75, 1⋅26)	2282 (24⋅6)	0⋅92 (0⋅75, 1⋅13)	1030 (11⋅1)	1⋅74 (1⋅20, 2⋅52)
Overweight	8355	1278 (15⋅3)	0⋅99 (0⋅76, 1⋅28)	1883 (22⋅5)	0⋅81 (0⋅65, 1⋅00)	1066 (12⋅8)	1⋅85 (1⋅28, 2⋅68)
Obese	2727	401 (14⋅7)	0⋅98 (0⋅74, 1⋅29)	643 (23⋅6)	0⋅84 (0⋅67, 1⋅05)	397 (14⋅6)	2⋅03 (1⋅39, 2⋅98)
Smoking							
No	16 048	2263 (14⋅1)	1	3988 (24⋅9)	1	2032 (12⋅7)	1
Yes	4819	836 (17⋅3)	1⋅12 (1⋅01, 1⋅24)	962 (20⋅0)	0⋅77 (0⋅70, 0⋅84)	495 (10⋅3)	0⋅90 (0⋅79, 1⋅01)
Drinking							
No	16 155	2298 (14⋅2)	1	3931 (24⋅3)	1	2119 (13⋅1)	1
Yes	4712	801 (17⋅0)	1⋅09 (0⋅98, 1⋅22)	1019 (21⋅6)	0⋅87 (0⋅79, 0⋅96)	408 (8⋅7)	0⋅66 (0⋅57, 0⋅75)

a Adjusted OR was estimated with consideration of the family history of hypertension, family history of diabetes, hypertension, diabetes, and abnormal lipid profiles in multivariate logistics regression models.

b Meat refers to either red meat or white meat.

c Body weight status was categorized based on recommendations for Chinese adults using body mass index.

## Discussion

In this community-based nutritional study, we aimed to investigate the epidemiological characteristics of meat, vegetable, and fruit consumption, and further to examine the associations of socio-demographic attributes with the likelihood of meeting intake recommendation among urban and rural elders aged 60+ years in regional China. It was identified that either the intake frequencies or the proportions of participants who met intake recommendations of meat, vegetable, and fruit were not high, and all selected socio-demographic characteristics were significantly associated with the likelihood of meeting intake recommendation of meat, vegetable, and fruit among participants in the study.

It is really meaningful to make a comparison of findings in our study with those documented in a recent nation-wide survey using data derived from China Health and Nutrition Survey 2015 (CHNS-2015 survey).^(21,22)^ The consumption rate of meat (red plus white), vegetable, and fruit was 99⋅4, 99⋅9, and 96⋅3 %, respectively, among overall participants in our study. Each of them was higher than that (81⋅9, 99⋅5, and 39⋅7 %, separately, for meat, vegetable, and fruit) reported in CHNS-2015 studies.^(21,22)^ Interestingly, compared to the proportion of participants meeting intake recommendation of meat (19⋅7 %), vegetable (34⋅3 %), and fruit (5⋅8 %) in CHNS-2015, the corresponding figure was, separately, lower for meat (14⋅9 %) and vegetable (23⋅7 %), but much higher for fruit (12⋅1 %) in the present study.^(21,22)^

These inconsistent findings in our study and the CHNS-2015 survey might be explained by at least three main reasons. The first might be due to the different numbers of participants included in these investigations. In our study, the sample size was as large as 20 867, and participants were well representative of the entire elderly population in Nanjing municipality. However, CHNS-2015 survey was conducted within overall adults, not elders only, from fifteen provinces of China.^(21,22)^ Considering that just approximately 5000 elderly subjects derived from the overall adult participants in CHNS-2015, the sample population might not be representative sufficiently of the national elderly population. The second explanation might be that different methods were applied to assess food intake in our study and CHNS-15 survey. Consumption frequency under a typical situation was used to measure food intake level in our study, while three consecutive 24 h recalls were employed to gather the intake amount of food for participants in CHNS-2015 survey.^(21,22)^ The third was that different approaches were used to assess an individual to meet food intake recommendation in the two studies.^(31,35)^ In our study, consumption frequency was applied to determine a participant reaching the recommended food intake level based on dietary guidelines specifically for elderly Chinese residents released by Chinese Nutrition Society in 2016,^(31)^ while the CHNS-2015 survey used consumption amount to judge a subject meeting food intake recommendations according to dietary guidelines for overall Chinese residents issued by Chinese Nutrition Society in the same year.^(35)^

Interestingly, the findings on associations of meat, vegetable, and fruit consumption with potential influencing factors in our study stratified by either of age, gender, and residence area (urban *v.* rural) were surprisingly in line with those documented in CHNS-2015 survey, although consumption frequency and intake amount of food were used, respectively, to assess food intake level in these two studies.^(21,22)^ Those older elders were less likely to meet recommended intake standard for meat, vegetable, and fruit, separately, compared to their younger counterparts. Women were at lower odds for reaching recommended standard of meat or vegetable consumption relative to men, while there was no difference in fruit intake between women and men. Urban elders tended to consume each of meat, vegetable, and fruit at recommended standard than rural elders. These consistent findings for sub-groups of participants from different studies with different food consumption measurement approaches imply that similar dietary patterns regarding meat, vegetable, and fruit consumption may hold for elders across China.

This study had several strengths. Firstly, the sample size was as large as about 20 000, and participants were randomly selected from both urban and rural areas. The study sample subjects were representative of overall approximately 1⋅5 million elderly residents in the megacity. Secondly, validated instruments were applied to assess food intake for participants. Thirdly, food intake recommendations specifically for Chinese elders, not overall adults, were used to assess participants’ meat, vegetable, and fruit intake levels. Finally, the scenarios for overall and stratified participants were well analysed and presented. Interesting findings were observed and could be used to inform precision healthy eating promotion campaigns with consideration of elders’ socio-demographic characteristics.

Although this is the first study on meat, vegetable, and fruit intake among representative elders from an entire typical megacity in China, several limitations shall be mentioned. First, information on food consumption was self-reported by participants, which might cause potential recall bias, especially for those older elders. Second, consumption frequency was used to measure subjects’ food intake level. This could not allow us to assess the intake amount of meat, vegetable, and fruit for participants. Third, the patterns of meat, vegetable, and fruit intake were analysed for Chinese elders using data from a single cross-sectional survey, which could not allow us to further investigate trends/shifts in the dietary intake over time. In the future, data from longitudinal or several repeated cross-sectional studies are welcome to assess trends/shifts in the dietary intake for elderly residents in China.

From the public health perspective, it is in need to initiate precision healthy eating campaigns for improving the health conditions of elders in a rapid-ageing society. For an individual, eating behaviours may change under different situations or with time going on, while population-level dietary patterns may also change with economic growth in a society. Therefore, precision dietary intervention programs for elders shall be developed only based on periodical assessment of specific food consumption patterns. In future, dynamic population-level studies are encouraged to investigate intake patterns of foods like meat, vegetable, and fruits among elderly people.

In conclusion, the intake frequency and the proportion of participants meeting intake recommendation of meat, vegetable, or fruit were not high among elders in regional China. Socio-demographic characteristics were associated with intake recommendations of meat, vegetable, and fruit. It has public health implications that participants’ socio-demographic characteristics and behaviours shall be considered for precision intervention on meat, vegetable, and fruit consumption in population-level healthy eating campaigns among elders in China.
